# High-throughput virtual screening for organic electronics: a comparative study of alternative strategies

**DOI:** 10.1039/d1tc03256a

**Published:** 2021-09-16

**Authors:** Ömer H. Omar, Marcos del Cueto, Tahereh Nematiaram, Alessandro Troisi

**Affiliations:** Department of Chemistry, University of Liverpool Liverpool L69 3BX UK a.troisi@liverpool.ac.uk

## Abstract

We present a review of the field of high-throughput virtual screening for organic electronics materials focusing on the sequence of methodological choices that determine each virtual screening protocol. These choices are present in all high-throughput virtual screenings and addressing them systematically will lead to optimised workflows and improve their applicability. We consider the range of properties that can be computed and illustrate how their accuracy can be determined depending on the quality and size of the experimental datasets. The approaches to generate candidates for virtual screening are also extremely varied and their relative strengths and weaknesses are discussed. The analysis of high-throughput virtual screening is almost never limited to the identification of top candidates and often new patterns and structure–property relations are the most interesting findings of such searches. The review reveals a very dynamic field constantly adapting to match an evolving landscape of applications, methodologies and datasets.

## Abstract 2

We review how high throughput virtual screening can been used to navigate the landscape of materials for organic electronics.

## Graphical abstract

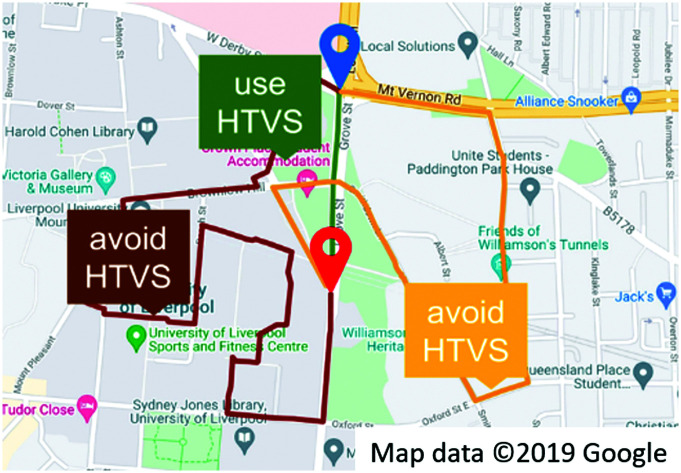

## 1. Introduction

The vision of employing organic materials as active components of electrical or optical devices, put forward and very actively pursued from the 80s^1^ has been remarkably fruitful with a range of products that have reached the mass market, like organic light-emitting diodes (OLEDs),^2^ prototype devices that approach their more established competitors, organic photovoltaic (OPV) devices,^3^ and components of flexible electronic devices like conductive inks.^4^ The field has been able to renew itself and identify new challenges, such as the development of novel emissive materials (dual emission,^5^ room temperature phosphorescence,^6^ thermally activated delayed fluorescence (TADF)^7^), the exploitation of multiexcitonic states (singlet fission^8^ (SF) and up-conversion^9^) and the application into novel domains like organic bioelectronics,^10^ neuromorphic^11^ and quantum computing.^12^ The premise for the successes and the optimism about the new challenges ahead is that organic materials for electronics can be fine-tuned with exquisite precision to have the desired electronic characteristics and the processing characteristics required for fabrication. While there is a substantial component of chemical fine-tuning in the progress of these fields, the greatest advances coincide with the introduction of novel material classes or model materials (*e.g.* semicrystalline polymers,^13^ solution-processable high-mobility crystals,^14^ n-type polymeric semiconductors^15^). Such breakthroughs have been historically the result of very extensive labour and an understanding of the physical principles, which only became more consolidated recently.^16–18^

True exploitation of the power of organic synthesis in electronic materials comes with the availability of reliable models that allow realistic prediction of properties, and it is therefore not surprising that computational modelling has accompanied the development of organic electronics throughout.^19^ The ambition to guide the discovery of new materials and contribute to innovative breakthroughs is instead more recent^20–22^ and has been promoted by (i) the availability of low-cost computational infrastructure, (ii) the facility of access to cheminformatics tools and databases, (iii) the increased robustness of quantum chemical methods and (iv) the penetration of data science methods in chemical and materials discovery.^23^ For the purpose of this work we can define high-throughput virtual screening (HTVS) as the computational investigation of a large set of compounds or materials to assess their suitability for a particular function. By “large”, here we simply mean sufficiently large to prevent the human inspection of the individual instances and requiring independent statistical validation of the accuracy of the procedure and automatic analysis of the output.

**Fig. 1:**
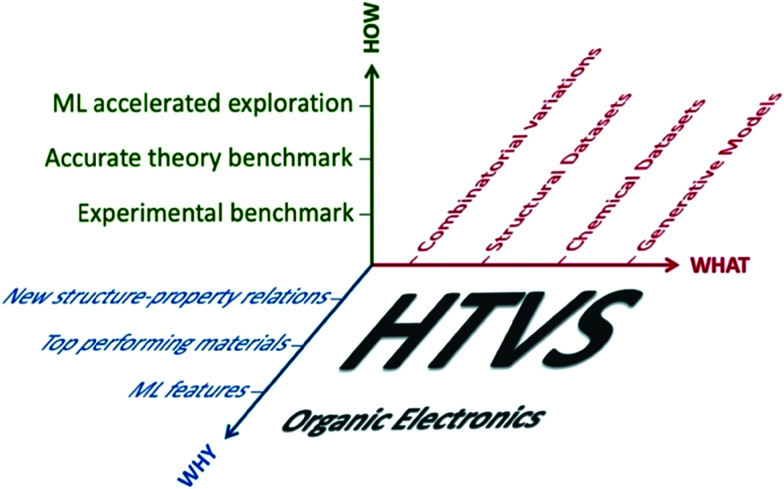
The dimensions of HTVS in organic electronics explored individually in this review. How to perform and validate HTVS (Section 2), what datasets can be explored (Section 3) and what questions can be answered (Section 4).

A survey of the literature reveals that works reporting HTVS of organic materials address the same series of questions (what can be computed, how are the results validated against experiment, how can one generate a dataset of candidates to screen and how can the results be interpreted or used). For each specific problem, one encounters multiple bifurcations and, as a result, there are almost limitless ways to perform HTVS for the very same problem (see scheme in Fig. 1). However, most of these choices are independent of each other and it should be possible to define an optimal workflow by analysing each component independently and adopting the best practice of each of them. The importance of developing more standardised workflows can be appreciated from Table 1, which reports a non-exhaustive list of organic electronics technologies and specific properties that require the identification of specialised molecular materials (alongside a representative bibliographic reference with more detail on the technology). The growing number of applications and the variety of requirements from different technologies suggest that HTVS of organic materials will continue to accompany this research field for many years to come and a standardised framework to analyse and improve HTVS would be very beneficial.

**Table 1:** Non-exhaustive overview of the technologies in organic electronics requiring the development of novel molecular materials with a sample reference providing an example of research work focused on the identification of the promising molecules for that function

Technology area	Property or function	Sample ref.
Organic/hybrid photovoltaics	Electron acceptors	34
	Electron donors	35
	Singlet fission	36 and 37
	Up-conversion	38
	Hole-transporting materials	39
	Sensitisers	40 and 41

Displays	Light emitters (IR & vis) for OLEDs	42 and 43
	TADF	44 and 45
	Dual emission	46

Lasers/probes	Materials for gain medium	47 and 48
	Deep-tissue optical imaging	49

Transistors	High mobility materials	50
	Biomaterial devices	51
	Phototransistors	52

Detectors	X- and gamma-ray	53
	Artificial vision	54 and 55
	IR detection	56

Sensors	Gas sensors	57 and 58
	Biological sensing	59

Memory	Resistive memories	60 and 61

Energy storage	Nonaqueous redox flow batteries	62
	Organic electrodes	63 and 64
	Pseudo-/super-capacitors	65 and 66

Photocatalytic systems	Organic photocatalysts	72 and 73
	Dye-sensitised photocatalysis	74

Others	Conductive inks	67
	Spin-valves	68
	All-printed RFID tags	69
	Neuromorphic devices	70 and 71

For these reasons, instead of describing, one by one, the main contributions in the area, we organise this review following the questions that should be answered in planning and performing HTVS and, specifically, what properties can be computed and with what accuracy (Section 2), how the datasets to explore can be generated (Section 3) and how the results can be analysed (Section 4). In this work, we will try to highlight areas where a consensus on the methodology is being reached and where contrasting approaches have been proposed. This deconstruction of the field of HTVS in organic materials should be seen as complementary to other reviews of the field.^24–26^ While the same questions are posed in search of inorganic materials, this review will not focus on them, and the reader is referred to other works.^27–33^

## 2. Computable properties: benchmarking and calibration

Although hardware is developing rapidly, the most accurate theoretical methods can be unfeasibly expensive, meaning that approximate methods are vital, especially for HTVS. This implies that benchmarking is often a necessary pre-step for HTVS, whether it is done by the researchers carrying out the screening or provided by other benchmarking studies in the literature. There have been multiple recent works^75–79^ with the purpose of accelerating the future computation of a specific property by comparison, modification, and combination of existing theory.

**Fig. 2:**
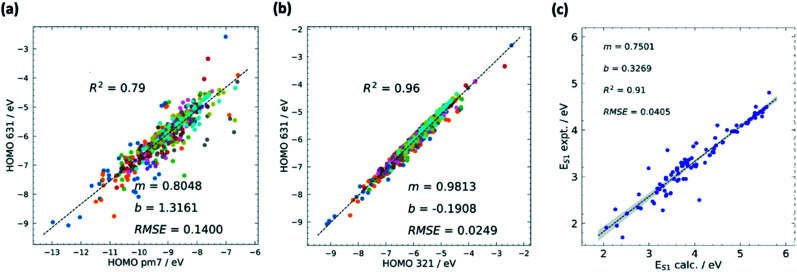
Calibrations in HTVS has different forms. The most common is the calibration of the lower *versus* the higher level of theory. The comparison between semi-empirical and DFT theory is often quite satisfactory and the comparison of results with different basis sets is typically excellent. (a) Example of calibration of HOMO energy level computed using semi-empirical (PM7) and DFT (B3LYP/6-31G*) methods with squared correlation coefficient *R*^2^, root mean squared error (RMSE), and the m and b values for the linear fit *y* = *mx* + *b*. (b) Calibration of HOMO energy level computed using DFT with different basis sets B3LYP/3-21G* and B3LYP/6-31G*. A combination of methods at different accuracies can be used sequentially in multilayer screenings. Comparison against experiment, often carried out with one of the best methods, highlights the systematic errors and the maximum accuracy achievable when this is corrected. (c) Comparison between calculated (M06-2X/def2-TZVP) and experimental values of first excited state energy *E*_S1_. Adapted from ref. 37.

Benchmarking is a way to find a cost-effective methodology to successfully carry out HTVS within the limitations of both project time and the hardware available. To properly benchmark, a small and diverse set of the larger input set can be evaluated by employing different protocols, *e.g.* for density functional theory (DFT),^80^ this could include a wide range of exchange–correlation functionals and basis sets. If experimental data for the molecules used are available, each of the methodologies can be assessed in terms of their correlation and error to experiment to figure out the maximum accuracy that can be achieved. As it is likely that the best methods may still be too computationally expensive for HTVS, the most efficient method can be chosen judiciously according to specific project constraints. The method can also be subsequently calibrated by identifying a simple relation (often linear) between computed and experimental data which reduces the systematic error of the calculation (see also Fig. 2). This is only available for screening studies that seek to evaluate directly computable properties such as optical absorption for single molecules where good and homogeneous datasets^81,82^ are available.

If there is no available experimental data, comparison with the most accurate (but time-consuming) methods, such as coupled cluster (CC) theory,^83^ can be done in the same way. The ultimate goal is to find a method which evaluates the property of interest to a good degree of accuracy, whilst also being computationally feasible for HTVS. In their extensive assessment of functionals to accurately calculate the singlet excited energy using TD-DFT,^84^ D. Jacquemin *et al.* suggest that although benchmarking to experiment is a logical philosophy to realise more accurate theoretical results, it is limited by both the uniformity, or lack thereof, of experiment between datasets, and the weakness of not being able to strongly mimic experimental conditions computationally, such as solvation effects. It is especially palpable for excited state computations, whereby only vertical excitations are normally considered; calculations of the more realistic adiabatic energies require further geometry optimisations on the excited state geometries, which is mostly limited to the higher-level refinement stages of a large-scale screening scenario. Benchmarking and calibration to higher levels of theory, *e.g.* state-of-the-art wavefunction methods, remove these conditions and grants uniformity to all reference datasets. In cases such as calibrating classical or semi-empirical methods to DFT, the size of reference datasets can also become substantially large, making the fits potentially quite predictive.^85–88^ Although the disadvantage of using theory to calibrate theory is clear, its successful applications have allowed it to readily percolate through the field.

Experimentally accurate post-Hartree–Fock *ab initio* methods, *e.g.* CCSD(T) are often the gold standard for calculating physical properties but, due to their very unfavourable computational scaling with system size,^89^ they cannot be used for HTVS of organic electronics. Despite the increased accuracy of intensive wavefunction methods, benchmarking of functionals and basis sets using (TD-)DFT can be enough to reduce the error in the computation of physical properties to acceptable levels for screening.^75,84,90–92^ A more available hierarchy is the use of DFT to fit results calculated using semi-empirical methods. This is especially useful when polymeric structures are being considered,^93–97^ as the system sizes are too large to evaluate with DFT directly, unless the expensive integrals can be parametrised.

To successfully benchmark and calibrate the output of a computational method, the property in question must be directly computable, *e.g.* ionisation potential (IP), electron affinity (EA), excitation energy *etc.* Properties which are only indirectly computable, *e.g.* SF yield or power conversion efficiency (PCE) and are not based on the outcome of a single calculation, cannot be treated in the way described above. The validation of directly computable properties is discussed in the next two sections.

### a. Frontier orbital energy and redox potential

A common focus of HTVS is on the evaluation of ground state properties such as the frontier orbital energies. The HOMO and LUMO energies are especially useful in calculating IP and EA which are often defined in vacuum. For many organic electronics applications (organic semiconductors (OSC), hole transporting materials, *etc.*), the energy level alignment of these values across the device is an essential prerequisite for their function. A range of experimental data have been developed over the years to validate the computation of IP and EA, with inclusion of larger, more relevant molecules for organic electronics taking place more recently. In terms of dedicated databases, the G2-97 test set consists of 88 experimental IPs and 58 EAs of small molecules,^98^ and is a collection of measurements with a reported uncertainty of less than 0.05 eV. The NIST Chemistry Webbook has thousands of IP and EA entries for small to medium sized molecules collated from literature sources.^99^ The method of measurement and uncertainty (which can be as high as 1 eV) is reported for each entry. In general, photoemission spectroscopy can provide the most reliable results for these properties.^100^

The computation of these quantities has been long assessed and improved with the various quantum chemical methods. For example, evaluating IP and EA using Koopmans’ theorem^101^ is crude if Kohn–Sham (KS) DFT is employed and can yield large errors, especially for EA.^92^ However, the error can be significantly alleviated if long-range corrections are introduced due to better treatment of fractionally occupied orbitals.^102–108^ Further investigations^109–118^ into the expensive GW^119–122^ approach shows improvement against KS DFT, however there is found to be significant improvement when long-range corrected hybrid functionals are used in tandem with GW corrections.^108,123,124^ Electron propagator methods^125–129^ are an additional way of finding IPs and EAs with good accuracy to highly-correlated wavefunction methods,^130,131^ while remaining, however, still too computational expensive for HTVS. This then leaves the various flavours of DFT, or more approximate methods, as the level of approximation used for the initial stage(s) of virtual screening for suitable orbital energy levels.

In order to use DFT for HTVS seeking to calculate IPs and EAs, it should be benchmarked for optimal accuracy. In ref. 92, a diverse set of 11 DFT functionals is assessed by validating against experimental IP and EA values in vacuum. They showed that the HOMO energy can be evaluated relatively accurately *versus* experiment for 27 common small molecules. Despite a systematic upward shift in the calculated energy, linear calibration with the experiment can yield predictive HOMO values, especially when functionals including exact exchange are used, such as KMLYP^132^ with *R*^2^ = 0.99 and an average error of 0.73 eV. This is also seen in a larger-scale study by Y. Fu *et al.* where 270 experimental IPs are compiled from the literature to calibrate the B3LYP^133,134^/6-311++G(2df,2p)//B3LYP/6-31+G(d) protocol; a strong linear dependence is found with *R*^2^ = 0.99 and a systematic error of 0.28 eV,^135^ indicating the existence of robust measurements for this property. The LUMO eigenvalue calculated in ref. 92 is, unsurprisingly, found to be extremely inaccurate in comparison to experimental values (*R*^2^ ≈ 0). As the calculation and experimental measurement of the gap (*R*^2^ = 0.91–0.96) and HOMO energy (*R*^2^ = 0.94–0.99) are shown to be more robust, it was proposed that the LUMO accuracy can be increased by subtracting the HOMO energy eigenvalue from the gap energy when using these types of methods.^92^

There are several screening studies,^63,136^ including large-scale,^137^ which evaluate IP and EA using DFT. For example, M. Korth studied 23 000 known small molecules for IP and EA.^138^ Their pre-screening work used the Koopmans approach to assess the accuracy of different quantum chemical methods on IP and EA, with benchmarking against the G21EA and G21IP subsets of the GMTKN24 database.^139,140^ Out of their tested DFT methods, PBE^141^/TZVP^142^ had the lowest error with mean absolute deviation (MAD) both for IP (MAD = 4.64 kcal mol^−1^) and for EA (MAD = 7.00 kcal mol^−1^).

It is, however, important for the discovery of new organic electronics that molecules of relevant sizes are also included within the benchmarking. In a multipart study,^108,123,131,143^ the vertical IPs and EAs of acenes, quinones, nitro/nitriles, anhydrides and other electron acceptor structures with experimental measurements from the NIST were evaluated with the best theoretical methods, *i.e.* within ±0.03 eV accuracy of CCSD(T) with complete basis set limit (CBS) extrapolation. The latter, regarded as the gold standard method and unfeasible for larger molecules, is expected to be near the Born–Oppenheimer *ab initio* limit, and major errors *versus* experiment (≥0.1 eV) are thought to be due to nuclear relaxation and environmental effects. This set of 24 known acceptors then forms a homogeneous, high-level reference for calibration of more approximate methods.

An additional step to increase the accuracy of IP and EA calculations, *versus* simply taking the negative of the respective orbital eigenvalue, is to calculate the difference between the total electronic energy of the neutral state and the charged state of the molecule. Furthermore, one can consider computing the adiabatic rather than the vertical transition energy, *i.e.* allowing for the nuclear relaxation of the charge state at the cost of an additional optimisation. It was proposed that this latter effect could be neglected in screening,^138^ as it produces larger errors only in few cases.^144^ The Electrolyte Genome Project by X. Qu *et al.*^145^ included nuclear relaxation effects in their automated and benchmarked high-throughput DFT screening. They sought to ensure the high fidelity of their results by evaluating adiabatic IPs and EAs with the inclusion of the solvation model IEF-PCM.^146^ Extensive protocols such as this can be reasonable with smaller scale and lower-level screening efforts.

Additional challenges with respect to the calculation of IP and EA are provided by the accurate evaluation of redox potentials which is essential in, for example, developing new materials for use in organic redox flow batteries and other devices.^137,147^ Solvation energy plays a major role in determining the redox potential and must be carefully assessed^147^ (this also means that insoluble compounds cannot be measured). Furthermore, some organic compounds suffer irreversible coupling of their monomers and can create additional difficulty in providing reliable values.^148^

An effort by H. Neugebauer *et al.*^149^ assessed the performance of the previously developed semi-empirical theory (GFN-xTB),^150,151^ PMx,^152,153^ and DFT for the calculation of redox potentials for small to medium sized organic structures. Their test set consisted of 193 organic molecules with experimentally determined redox potentials,^154,155^ allowing for a relatively robust evaluation of each method. They showed that DFT is bottlenecked by inadequate treatment of solvation effects (MAD = 0.22 V/*R*^2^ = 0.97 for their best method: PWPB95-D4^139^/def2-QZVPP^156^). This finding is supported by a protocol comparison study by M. Isegawa *et al.*, which showed a similar error in the redox potentials for even the highly-correlated CCSD(T) method.^157^ Their tight-binding based method (GFN2-xTB) was comparable with DFT (MAD = 0.30 V/*R*^2^ = 0.94), whereas the PMx class of semi-empirical methods were poorer approximations (MAD ≈ 0.60 V/*R*^2^ ≈ 0.88). Ultimately, their novel tight-binding methodologies were approximately 2–3 orders of magnitude faster than the DFT counterparts, and around one order of magnitude faster than the PMx protocols. What is then left for further improvements in the evaluation of redox potentials is related to the improvement of solvation models, and work has been done to validate both explicit solvation,^158^ and the effect of considering a diverse range of compounds, rather than picking a homologous set which is known to work well with an empirically parametrised model.^159^

### b. Excited state energies

To be able to study interesting photophysical phenomena such as fluorescence,^160,161^ phosphorescence,^162,163^ SF,^164–167^ TADF,^168–171^*etc.*, one must consider the electronically excited states of a structure. There are a range of experimental datasets available to validate a theoretical evaluation of these energies, particularly S_1_ and T_1_. This is largely due to the ease of the experiments, *e.g.* UV-vis, absorption and luminescence. Most molecules maintain a relatively constant electronic structure when the solvent differs between measurements, unless some of the relevant states have a strong charge transfer character,^172^ the transition energies are only moderately affected by solvent effects, making it easier to construct large, reliable experimental datasets to be used for benchmarking and calibration. It should be noted, however, that experimental energies of low triplet states may require less straightforward measurements at low temperature or in polymeric matrices^173^ making the availability of data more limited in certain spectral regions.

The extensive TD-DFT benchmark in ref. 84 constructed an experimental training set of 483 molecules from literature sources.^174–182^ Using this dataset, they assessed the qualities of 29 different functionals on predicting excited state transition energies. It is found that pure functionals systematically underestimated the energies with mean absolute error (MAE) of approximately 0.35–0.4 eV whereas mixing in some exact exchange, optimally 22% to 25%, reduced the MAE to around 0.26 eV and 0.23 eV, as with the TPSSh^183^ and B3LYP^133^ functionals respectively. Moving from pure functionals to those with fractions of exact exchange also increased the correlation between the theoretical and experimental values, *i.e.* 0.94 to 0.96, which allowed for a more confident calibration, and indeed, combination of the best results through multiple linear regression^184^ pushed the *R*^2^ correlation as high as 0.98.

The Handbook of Photochemistry contains the S_1_ and T_1_ energies of approximately 500 organic molecules;^81^ one such study^37^ which used this data to calibrate a previously benchmarked protocol^185^ is an HTVS done by our own group to find SF active molecules existing within the Cambridge Structural Database (CSD).^186^ The protocol benchmarking study in ref. 185 assessed the quality of local hybrid functionals for the evaluation of S_1_, T_1_ and T_2_ energies, with particular emphasis on the T_1_ state due to its potentially poor description by TD-DFT.^187^ They used a small test set of 11 SF candidates previously calculated with CASPT2,^188^ however updated this reference level to CC2/CBS for better accuracy uniformity since CASPT2 can be sensitive to the selection of active space orbitals.^189^ It was found with the best protocol which combines TD-DFT for, S_1_ and T_2_ and ΔSCF for T_1_ (Lh12ct-SsifPW92/def2-QZVPD//BLYP35/def2-TZVP) that the MAE for the vertical S_1_ and T_2_ energies were around 0.10 eV, and just over 0.20 eV for the T_1_ energy. In the following screening effort in ref. 37, the more widely available and similarly accurate M06-2X functional was adopted with the basis set lowered to def2-TZVP for all stages. Using 100 S_1_ and T_1_ energies of small to medium organic molecules from the reliable experimental dataset,^81^ the root-mean-square deviation (RMSD) for the calculated S_1_ energy with the experiment was 0.0405 eV with *R*^2^ = 0.91, and RMSD = 0.0537 eV with *R*^2^ = 0.88 for the T_1_ energy. This accuracy allowed for the final calibration of results to identify molecules with the desired S_1_–T_1_ energy difference.

There are other experimental datasets available which are popular for machine learning (ML) techniques but can be used for benchmarking and basic calibration using many datapoints, for example the collection by J. F. Joung *et al.*^190^ includes the optical properties for over 7000 unique chromophores, ranging from small molecules to relevant molecules such as pyrene,^191^ coumarin,^192^ azobenzene^193^*etc.* However, many screening studies opt to use smaller sets of specialised molecules due to the availability of experimental data. This is usually done by searching through literature that includes experimental measurement, either by automatic or manual extraction, to construct a unique training set and benchmark reference for the screening. R. Gómez-Bombarelli *et al.*^42^ pieced together a training set of 46 molecules from other works^194–210^ to calibrate the results of their TD-DFT calculations in an attempt to find TADF active molecules, which are often large and complex in comparison to molecules existing in experimental datasets. Another study which followed this method is by N. M. O’Boyle *et al.*,^96^ where the primary excitation energy of 60 literature-based oligomeric compounds, taken from their previous benchmark study,^97^ was compared with experiment, yielding RMSD = 0.28 eV, and *R*^2^ = 0.84 when using the PM6/ZINDO^211^ method.

As an alternative to calibration against experimentally determined excited state energies, it is possible to compare with high-reference excited state calculations. If computed data for excited state properties are to be considered for calibration, there are very high-level reference datasets available.^212–217^ For example, the excited states, including triplets, of 18 small molecules, *e.g.* water, acetylene *etc.* were evaluated with up to the CCSDTQP level of theory, and almost reached full configuration interaction standard.^218^ With the expansion of the molecules considered, now encompassing 27 medium sized molecules such as benzene, thiophene, triazine *etc.*, ref. 219 provides data up to the CCSDTQ level of theory for molecules with 4 heavy atoms, and CCSDT for those with 5 and 6 heavy atoms. It is believed that, for the theoretical geometries, almost 95% of their reported transition energies are chemically accurate to 1 kcal mol^−1^, and the errors can be attributed to geometry differences and inconsistencies in measuring vertical transitions experimentally.

There are a number of very large datasets which contain theoretical reference data at more feasible levels, *e.g.* TD-DFT and are nominally used for ML-based studies. For example, VERDE Materials DB^220^ hosts 1500 organic electronic relevant structures with computed excited state energies at the M06/6-31+G(d,p)^221–223^ level. The QM7b^224,225^ database provides information on over 7000 structures based on the 7 heavy-atom subset of the GDB-13.^224^ The QM8^226,227^ includes 20 000 synthetically available small molecules, and provides electronic structure values using both TD-DFT and CC2. The QM-symex^228^ has 173 000 compounds with excited state energies calculated at the B3LYP/6-31G level, and provides particular emphasis on molecular symmetry. Such databases could be used for the calibration of lower-level methods, *e.g.* semi-empirical; this can be especially useful for the preliminary stages of HTVS projects where molecules are to be filtered prior to secondary stages. Works based on high-throughput screening of the excited state energies of polymeric structures could benefit most from this philosophy as the main bulk of the calculations will necessarily need to use low-level theory such as semi-empirical methods, with the references computed using TD-DFT, for example.

In contrast to time-dependent methods, the optical gap can be estimated using the frontier orbital energies. Testing on a set of benzofulvene derivatives, S. Tortorella *et al.* showed that the optical gap can be predicted reasonably well, in terms of correlation, by taking the HOMO–LUMO difference when using the ZINDO semi-empirical method on AM 1 optimised geometries (MAD = 3.01 eV/*R*^2^ = 0.83).^91^ Naturally, the higher level DFT methods such as B3LYP produced better results when compared with experiment (MAD = 0.26 eV/*R*^2^ = 0.87) especially in terms of the systematic error, though interestingly the same correlation could be achieved if the B3LYP geometry was used with a ZINDO electronic structure calculation (MAD = 2.74 eV/*R*^2^ = 0.87). This finding implies that, if the correlation is good, low-level theories such as semi-empirical methods can be used for rapid pre-screening of even millions of molecules, prior to higher level refinement, which is in line with the typical computational funnel hierarchy of HTVS. However, there should be careful consideration when these very approximate methods are used as they are often parametrised to reproduce experimental values for specific structural motifs, and can fail to generalise.^229–231^

The use of semi-empirical methods, such as ZINDO, AMx,^232–235^ PMx and so on^236^ to compute optoelectronic properties, *e.g.* band gap, of conjugated polymers has been a focus of screening studies instigated by G. R. Hutchison *et al.* with more recent investigations into soft modelling approaches using genetic algorithms.^95–97^ In a similar vein, M. A. Zwijnenburg and co-workers used a semi-empirical high-throughput screening approach to calculate IP, EA, and the optical gap for conjugated polymers.^93^ They used the xTB methods developed by S. Grimme *et al.*^150^ and linearly calibrated with (TD-)DFT using a set of 40 copolymers; this, to an extent, allowed for (TD-)DFT accuracy for unknown polymeric structures computed with the xTB methods. They followed the same calibration approach to search for novel, diketopyrrolopyrrole based dyes for use in OPVs.^74^

Although there are datasets of organic molecular properties,^81^ the field still lacks the availability of a large set of data for crystalline properties except for reviews of selected topics^237,238^ from which it is possible to collate information on the optical spectra and luminescence of about ∼100 molecular crystals. This set is, however, sufficient to provide validation for the computation of the excitonic coupling^239^ which is one of the key elements needed for the study of solid-state optical properties. The other key element is the evaluation of the local exciton phonon coupling^240,241^ which is a property of the isolated molecule and is computable with a good accuracy.^242^

A general observation that can be made after the overview of the past two sections is that the calibration against experiments and higher-level theory for both excited and ground state properties is generally less systematic in works focused on organic electronic applications, *i.e. ad hoc* calibrations are often proposed to maximise the accuracy of the predictions within a given technological context. On the contrary, contributions directed toward the community of quantum chemistry have developed more standardised datasets for testing (albeit generally based on smaller molecules). To better track future progress and strike the right balance between accuracy and computational cost it would be ideal to develop common datasets that sample a broad range of compounds used across organic electronics.

### c. Charge mobility

Charge carrier mobility is one of the main figures of merit used to determine the suitability of organic semiconducting materials for technological applications. Molecular semiconductors have been considered traditionally more approachable for benchmarking and HTVS studies as, unlike polymers or amorphous materials,^16,243,244^ there are no unknown structural or morphological features to be determined. Charge mobility measurements of molecular semiconductors that are robust and reproducible in different research labs have started to appear relatively recently.^245^ The measured mobilities, however, depend on the purity of crystals,^246^ and the degree of polycrystallinity.^247^ Therefore, the intrinsic (defect/trap free) mobility of single crystals is the only reference experimental value to study the relation between crystal structure and mobility. Particularly valuable are therefore those reports of mobility with intrinsic nature of transport verified either by comparison with Hall mobility,^248^ or “band-like” temperature dependence (*i.e.* mobility decreasing with increasing temperature).^249^ Considering these conditions, there are approximately 20 “reference” measurements of intrinsic mobilities in thin-film transistors, which provide a limited, but reasonable, set of data to validate the theory.^250^

The success in intrinsic mobility measurements has led to an important advance of technologies based on organic thin-film transistors,^250^ but it has also challenged the theory of molecular semiconductors that was thought to be well-understood. It is evident that the measured charge mobilities of high purity crystals (>1 cm^2^ V^−1^ s^−1^) are too high to be rationalised with a simple hopping transport mechanism yet too low to be accurately consistent with the band transport.^251,252^ This is more clearly discussed in ref. 253 where through evaluating mobility of ∼60 organic crystals using both theories, it is shown that, at room temperature, neither of these transport theories can accurately predict the charge carrier mobility. In molecular semiconductors, the Hamiltonian parameters, including charge transfer integrals, vibrational energies, reorganisation energy, dynamic disorder, and thermal energy at room temperature, differ by not more than an order of magnitude, which makes the evaluation of charge transport a challenging task.^18^ Accordingly, over the years, a number of advanced theoretical methods such as small polaron theory,^254,255^ mean-field Ehrenfest model,^256,257^ trajectory surface hopping method,^258,259^ open quantum systems,^260,261^ quantum Monte Carlo^262,263^ and transient localisation theory (TLT)^264–266^ have been developed to evaluate charge transport in this materials class. Most of these methods, despite being promising in predicting mobility in agreement with experimental measurements,^267,268^ are slow and, hence, have never been employed in HTVS studies.

Hopping transport theory, despite being not fully applicable to molecular semiconductors, due to its simplicity and low computational cost, remains a frequent method of choice in the majority of HTVS studies, including those using large databases,^269–271^ as well as those evaluating smaller libraries of structures.^272–275^ Applying this theory to screen the CSD, as the world's largest repository of small organic/metal–organic molecules whose crystalline structures are experimentally known,^186^ C. Schober *et al.* made an important contribution demonstrating, for the first time, that large scale screening of materials for transport applications is possible.^269^ The transfer integral and the reorganisation energy are the main components of the hopping transport theory. The computation of these two quantities is not very demanding, and their validation against accurate theoretical methods and experimental data is straightforward. There are a number of different methods that can be employed to compute charge transfer integrals.^276^ Computed band structures can be validated against more accurate computational methods, *e.g.* larger basis sets in DFT-based methods, as well as experimental data such as those obtained from angle-resolved photoemission experiments (ARPES).^277–279^ The calculations of the reorganisation energies rely on the high vibrational frequencies where the routine DFT methods provide reliable results. The results of these studies have shown remarkable agreement with experimental data extracted from infra-red and Raman spectroscopy.^280–282^ In some of these hopping theory-based virtual screening studies, experimental verification of the mobility of the targeted molecules is also provided, which shows only a qualitative agreement with theoretical results.^283^

All the advanced theories developed for accurate calculation of mobility of molecular semiconductors, as described above, require the nonlocal electron–phonon couplings elements.^284,285^ However, the calculation of this parameter is known to be computationally very demanding. Recent calculations of nonlocal electron–phonon couplings on one^268^ or few^286,287^ molecules required millions of CPU hours^288^ for state-of-the-art methods (*i.e.* not suitable for HTVS) while computationally inexpensive empirical force fields yield inaccurate results as they are not parameterised to reproduce low-frequency phonons.^289^ From the experimental side, Raman spectroscopy^280^ and terahertz time-domain spectroscopy^290^ are the most common methods used to derive information on low-energy phonons. However, these methods provide only gamma phonon energy and, therefore, can only partially verify the computed phonon spectra.^286^ High-resolution inelastic neutron scattering measurement is shown to be a more useful method as it allows retrieving information on the low-energy phonons and recently has enabled the validation of low-frequency phonon calculations in the context of evaluating charge mobility of molecular crystals.^288^

The calculations of nonlocal electron–phonon couplings can be substantially accelerated using simplified methods. For example, our group suggested using approximate phonons, assuming that each molecule oscillates independently from the others, to significantly speed up the calculations and make them feasible for HTVS studies. Relying on this strategy and applying the transient localisation theory, which is among the advanced theories that consider all transport parameters on the same footing, the CSD was screened for high mobility materials.^50^ The absolute values of calculated mobilities in the framework of TLT are within ∼35% of experimental data, and the relative values are well reproduced in families of homogenous compounds.^265,288^ Furthermore, as shown in a recent study, this theory is also able to reproduce electromechanical responses.^291^ The fragment-orbital based surface hopping method, relying on explicit time propagation of the electron-nuclear dynamics, is another viable approach that is used to capture the impact of thermal fluctuations on charge transport.^259^ A recent study, through applying this theory to a set of eight crystalline structures, shows that the excess charge carrier leads to a polaron which is delocalised over 10–20 molecules in highly conductive crystals. Charge mobilities extracted in this study are in remarkable agreement with experimental measurements and correlate strongly with the data obtained using TLT. The comparison between experimental and computed charge mobilities exemplifies a common situation in organic electronics where benchmark experimental data are limited for some properties and the modelling tools are not yet fully optimised. In these cases, the great demand for high performing materials is driving both standardisation of experimental methods^245^ and refinement of the theories.^18^

### d. Interfacial properties

Many of the functions of organic electronics materials are acquired at the interface with other materials^292^ and most organic electronic devices are fabricated as multiple layers of distinct materials^293^ or are based on multi-phase components with complex morphologies and often unknown composition.^294^ These aspects are at the forefront of current research and, since consensus is yet to be reached on the underlying physical mechanisms, they are not directly tackled by HTVS. The virtual screening in these cases often focuses on easily computable properties that capture only some of the physics. Examples are the search for alternative electron acceptor molecules for OPV that have similar electronic and solubility characteristics to the best performing ones,^295^ or the development of statistical models only considering energy levels of the constituent molecules.^34^

While it is and will remain impractical for many years to model many interfaces between soft organic materials with state-of-the-art methods,^296–298^ several authors have considered approximate models of the interfaces that allow their exploration in larger numbers. For example, Y Imamura *et al.*^299^ presented an approximated model to study the interfacial geometry of 1850 donor–acceptor pairs looking for arrangements more favourable to charge generations. Alternatively, one can build fewer models able to capture more physical details, like the proposal by C. Poelking *et al.* to model the electrostatics of bilayer cells which explain, very well, a range of measurements and could be included into future screening protocols.^300^ A type of interface that is amenable to HTVS is that between crystalline phases and chemisorbed molecules found, for example, in dye sensitised solar cell. In these cases it is possible to separate the role of the anchoring group from that of the rest of the dye^301^ and screen separately for the best anchoring group^302^ and the best dye.^40,74^

### e. Crystal structure prediction

For a range of organic electronics applications, the function is determined by molecular arrangement in highly ordered or crystalline domains, *i.e.* the properties are a combination of molecular properties and intermolecular interactions. Very substantial progress has been made in the field of molecular crystal structure prediction (CSP), *i.e.* the prediction of the molecular arrangement in the crystal starting from the chemical topology of the constituent molecules. This research area, more commonly employed in drug discovery and fundamental research, is reviewed often^303–305^ and we focus here on its recent ramifications in the field of organic electronics.

At the moment, it is not possible to perform HTVS where the starting point is a large set of molecules, and the output is their predicted crystal structure and the property of the material. With progress in sampling methods, adopting efficient, accurate atom–atom force fields, and employing parallel, high-performance computing, the situation seems to be changing.^303,306^ For instance, a library of 27 structural isomers of pyrido[2,3-*b*]pyrido[3′,2′:4,5]pyrrolo[3,2-*g*]indole is screened to assess charge mobility in their predicted crystal structures.^307^ This study yields two molecules with desirable charge mobility, which are also attractive as synthetic targets. A follow-up study from the same research group, using an evolutionary method, has screened a larger user-specified region of chemical space, containing aza-substituted pentacenes, to identify high-mobility materials.^308^ The first step of this analysis realises a set of promising molecules which are eventually evaluated using crystal structure prediction. Results reveal two promising structural motifs: aza-substituted naphtho[1,2-*a*]anthracenes having reorganisation energies comparable to that of pentacene and a series of pyridazine-based molecules possessing both low reorganisation energies and high electron affinities. Similar methods have been applied to classify molecules capable of forming weakly bound polyaromatic hydrocarbon co-crystals.^309^ As such, a two-step approach is developed that first utilises all the known molecular combinations forming this class of co-crystals, extracted from the CSD (1722 molecular combinations), to train the model. Then, in the second step, it ranks possible, but unknown, pairs from the ZINC15 database^310^ (21 736 possible molecular combinations). The applicability of the employed methodology and discoveries are verified through the experimental realisation of two co-crystals named pyrene-6*H*-benzo[*c*]chromen-6-one and pyrene-9,10-dicyanoanthracene both comprising molecules never considered as co-crystallising in the CSD.

### f. Conformational search

Conformational search is an important aspect of computational chemistry established in the field of drug discovery. Many drug molecules possess a large number of rotatable bonds,^311,312^ and it is often not enough to consider only a single conformational isomer. Although extending this idea into the field of organic optoelectronics may appear to be an unnecessary step since the chemical space is made up of rigid, conjugated structures and the free rotation of flexible side chains tend to have little effect on the electronic structure of an isolated molecule,^313,314^ it has been found that its impact can be far from negligible.^315^ It is especially true for functionalised organic electronic materials which contain rigid oligomers connected formally by a single carbon–carbon bond. This architecture allows for large flexibility on the dihedral angle between the monomers which, often modulated by steric effects,^316,317^ can generate a range of conformers with different properties.^318^ Consideration of a suitable conformational search step prior to HTVS is now considered necessary to ensure optimal accuracy. Works on polymeric structures^93,95^ are especially reliant on a good conformational search strategy to achieve higher accuracies. A common method between these studies is to generate a (large) sample of conformers using molecular mechanics (MM) methods at a low cost to find the lowest energy conformer. This conformer is then selected and subject to higher-level computations, rather than a randomly generated or distorted (higher energy) orientation.

Recent considerations by G. R. Hutchison *et al.* showed that finding the lowest energy conformer with MM methods may offer a poor starting geometry when compared to more accurate, quantum chemical methods.^319^ In other words, a consequence of using the lowest energy MM conformer prior to screening is that a high-energy geometry will persist with subsequent optimisations and will not reflect the geometry found if quantum chemical methods are used within the conformational search step. The difficulty in incorporating conformational effects in HTVS is particularly severe while modelling charge transport properties in polymers. There is a consensus on the dependence of transport on the local ordering of the polymers^320^ and a range of accurate works demonstrating how the local structure requires extensive molecular dynamics simulations,^321^ which are inconsistent with high-throughput screening. There is however some preliminary evidence that the conformational study of isolated chain coupled with electronic structure calculations can still provide sufficient guidance for the design of new materials.^322^

### g. Acceleration of HTVS by machine learning

Machine Learning (ML) has proved to be a valuable tool to produce results with an accuracy similar to quantum chemistry methods at a fraction of their cost, given that one has enough data to train the model. For an in-depth review of this field, we refer the reader to ref. 323 and 324. Here, we will focus on how the recent developments of ML impact the computation of properties of interest for organic electronics.

**Fig. 3:**
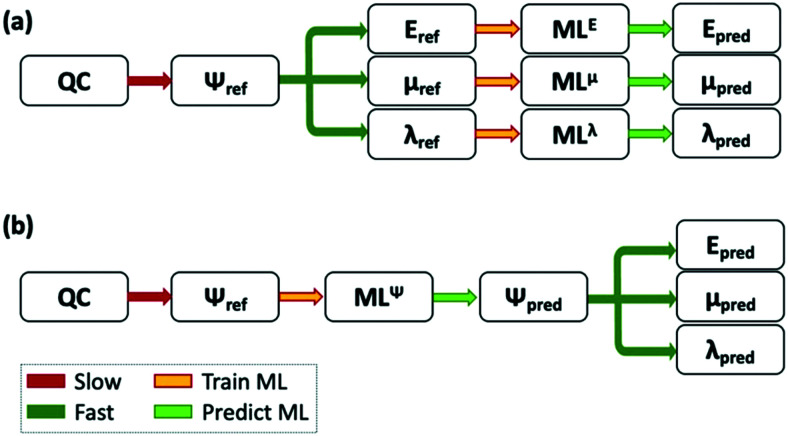
Scheme of the use of ML to complement QC calculations. (a) A ML model is trained to predict each feature. (b) The ML model is trained to predict the wavefunction of the system, and the model can then be used to predict multiple properties. Adapted from ref. 339, under a Creative Commons Attribution 4.0 International (CC BY) license.

M. Rupp *et al.* developed an ML model that calculates the optimisation energies from the nuclear charges and atomic positions of approximately 7000 organic molecules,^325^ and obtained an MAE of ∼10 kcal mol^−1^, similar to mean-field electronic structure theory at only a fraction of the computational cost. ML has also been used to correct the PM7 HOMO/LUMO eigenvalues and molecular polarisabilities (as well as other thermochemical properties less relevant for OPV applications) to an accuracy similar to DFT,^326^ where the ML correction represents only a fraction of the computational time of the semi-empirical calculation. This approach opens new possibilities and as data availability of quantum chemical calculations increases, the use of ML to improve and bypass new calculations will become more prevalent. ML has also been used to bypass DFT calculations,^327^ saddle point searches^328^ and force calculations during molecular dynamics.^329^ More recently, ML has been used to correct DFT atomisation energies to an accuracy of higher composite methods (G4MP2) with an accuracy of 0.005 eV for approximately 100 000 small molecules, and 0.012 eV for larger molecules with 10–14 heavy atoms.^330^ In a relevant application for organic electronics it has been shown how neural networks, trained with 200 000 molecules from the Harvard Clean Energy Project Database,^331^ can reproduce HOMO and LUMO results of quantum chemical calculations with a large level of accuracy.^332^ F. Jabeen *et al.* used a multi-linear regression analysis to predict the refractive index of a set of approximately 100 polymers.^333^ ML has also been used to predict the emission wavelength of multiple fluorescent organic molecules, using steric, hydrophobic and electronic properties.^334,335^ Other examples of the prediction of excited state properties through machine learning and a more general view on its applicability can be found in ref. 336 and 337. Alternatively, approaches have been proposed in which ML is used to approximate the wavefunction itself,^338,339^ and all properties of interest are extracted from it (see the difference with the more common approach of predicting each property separately in Fig. 3). An example of this approach was presented in ref. 340, where ML is used to approximate the atomic charges, instead of calculating the self-consistent charges during density functional tight binding calculations, which are then used to approximate the ground and excited state potential energy surfaces of multiple organic molecules.

These results show how ML can correct low-level calculations and approximate more expensive quantum chemical calculations for a variety of cases, and such models can be accurately trained with the order of thousands of training samples. However, one should also consider the lack of extrapolation capabilities of ML discussed for example in a recent work by S. Kauwe *et al.*^341^ The authors considered a dataset extracted from the Automatic Flow for Materials Discovery (AFLOW) database^342^ with a large variety of compounds with evenly distributed DFT properties, and they could consistently identify most of the materials with the 1% highest performing bulk modulus, thermal expansion and other properties, when training the model with the other 99%. Despite the encouraging results, the authors themselves indicate some limitations that can make this identification of highest performing materials not possible, *e.g.* when the high performance of the new material is due to a new physical mechanism not present in the training set, when datasets have imbalanced classes, lack heterogeneity or lack properties of interest, or when rare events cause drastic property changes. To address these problems, new metrics have been proposed to identify molecules outside the models’ domain of applicability,^343^ increasing the confidence of ML-guided exploration of the chemical space. Explicit quantum chemistry calculations can always be used to correct the erroneous predictions of materials with exceptional properties (false positives), which are typically in very small numbers. The greatest risk of ML methods bypassing direct calculations are the false negatives, *i.e.* exceptional materials not identified as such, which would be missed by the virtual screening.

## 3. Methods to sample the chemical space

Once the computational methodology for a target property has been determined and validated, the definition of the chemical space to be explored is the next major challenge of HTVS. In an HTVS, the generation of 3D structural information is often not a trivial task. The very first choice is whether the search for a novel property should be done among pre-existing compounds which are inherently synthetically available, or completely original chemical motifs. If pre-existing molecules are to be ignored, the chemical space of organic molecules, which is essentially impossible to sample fully (∼10^60^),^344,345^ is the vast sea which must be navigated. However, randomly generating molecules which simply follow the rules of organic chemistry is not an efficient way to search for niche phenomena, as this will quickly produce a large list of uninteresting structures and lends no consideration to the ease of synthesis. Synthetic accessibility of hypothetical compounds is arguably the most important aspect of *de novo* library design. If, for example, theory predicts a novel structure to have a higher PCE than any other known OPV, it is not useful if it cannot be synthesised; the only utility for such a case is the potential for accessible derivatives, or the development of new design rules. An alternative method is to use chemical motifs already known to exhibit the desired property as the chromophore bases for the input set.

A variety of approaches have been proposed that differ fundamentally in terms of dimensionality of the space explored, similarity of the molecules with respect to known examples and importance given on the chemical accessibility of the proposed candidates. Each approach is defined and exemplified in the following subsections.

### a. Combinatorial modifications

There are several ways to modify, enumerate, substitute, and combine chemical motifs in the pre-screening stages, and there have been multiple efforts developing increasingly intelligent algorithms to do this automatically, *e.g.* genetic algorithms can use physical and electronic data of chemical motifs to find useful combinations based on the screening criteria;^95^ they can also be used to find more accurate low-energy conformations for flexible molecules.^346^

Introduction of such algorithms is not always necessary to generate a rich input library. More simplistic approaches where fixed moieties are functionalised at random, or generic chemical formulae are filled in with relevant units have found purchase in the field of HTVS with reasonable success.^74,347–352^ The Harvard Clean Energy Project,^331^ for example, used 26 common building blocks, *e.g.* thiophene-, furan-, triazine- and benzene-based compounds with predefined connecting points to construct a set of 10 million unique molecules, including oligomeric sequences with up to five units. M. Moral *et al.*,^353^ on the other hand, took a more restricted approach and investigated 100 1,4-bis(phenylethynyl)-benzene derivatives with the formula Y–C≡C–X–C≡C–Y. This rigid architecture was used with consideration of the axial rod-like feature, which is prevalent in molecular scale electronics.^354–356^ With judicious choice of aromatic motifs in the X and Y position, the screening found structures which independently satisfied ambipolar charge transport characteristics, electron and hole transport, Ohmic contact with common electrodes and so on. Using a more exhaustive strategy like that in ref. 331, N. N. Matsuzawa *et al.* constructed a very large set of rigid acenes with two to eight fused rings and substituted with carbon, oxygen, sulphur and selenium.^270^ Since random permutation methods can quickly generate an unfeasible number of molecules, in this case over 7 M structures, a selection of a smaller subset is often necessary for the screening. The selection can be random, guided by some loose logic or by synthetic score. There are multiple ways to score the complexity or synthetic accessibility of molecules, these can range from expert opinion to neural networks, chemical similarity indices and structural descriptors based on graph theory.^357–359^ Y. Wen *et al.* considered synthetic accessibility in their DFT and ML combined approach to screen approximately 10 000 novel dyes for DSSC applications.^40^ For 500 promising hit candidates, they provide the synthetic accessibility score by Ertl and Schuffenhauer.^360^ They pointed out that some of the candidates with high predicted PCE are also predicted to have low synthetic feasibility. This work highlights the importance of considering synthetic feasibility; something that is often overlooked when *de novo* molecular generation is adopted for HTVS.

**Fig. 4:**
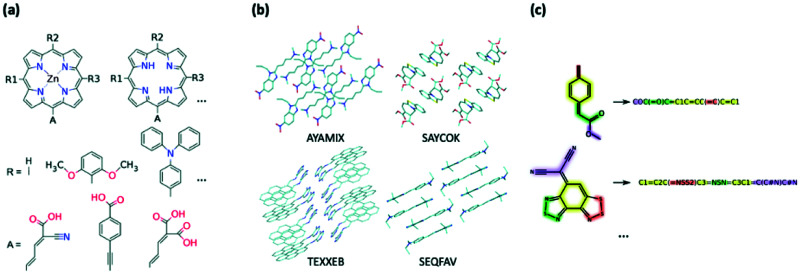
Example of generation of datasets for HTVP *via* (a) combinatorial modification of chemical motifs (from ref. 361), (b) structural dataset containing geometry and molecular crystal arrangements, (c) chemical dataset represented only through their chemical connectivity, *e.g.* SMILES strings.

Creating a collection of novel molecules can be done in a way which better facilitates the ease of synthesis by using stricter design rules or combining the existing moieties in a chemically logical way. There are screening efforts which follow this more intuitive approach to library generation; for example, K. B. Ørnsø *et al.* studied 1029 original, functionalised zinc porphyrin rings for DSSC applications and are generated by substitution of four peripheral positions (Fig. 4a).^361^ Their contained set of seven electron donors and three electron acceptors was driven by well-established knowledge of their properties and synthetic availability as they have been investigated experimentally or theoretically prior. A smaller-scale study by J. T. Blaskovits *et al.* looked at finding new SF candidates by pairing known donor and acceptor cores to create 81 novel copolymers.^362^ The constituent moieties were chosen to ensure short synthesis with high atom economy.

All of the efforts mentioned above used in-house techniques to modify existing motifs to generate an input set prior to computation of properties, and this may be the most reasonable approach when searching for a certain property, *e.g.* high PCE. It is also possible to generalise this process and consider all chemically plausible molecules that can be created as done in the GDB-13^224^ and GDB-17^226^ sets. These databases exhaust the chemical space of possible organic molecules with up to 13 and 17 heavy atoms respectively. Naturally, these projects have no prior consideration of synthetic accessibility but can be used to lead investigations into completely new design rules. R. Ramakrishnan *et al.* probed into this diverse, computer-generated chemical universe and provided the ground-state properties of a subset 134 000 structures from the GDB-17 using benchmarked DFT methods.^363^ There are other comprehensive works which also consider very large subsets, some with the inclusion of isomerism effects, and are invaluable for ML-based studies. For example, the ANI-1 database reports 20 million structures based on over 57 000 small organic compounds and their conformations.^364^ Using benchmarked DFT to provide more chemically accurate physicochemical results, ML algorithms are given a wealth of training data, including the fine structural differences. In a similar vein, the QM7-X set contains physicochemical data on approximately 4.2 million structures,^365^ including many isomers for improved application of ML techniques in drug design;^366^ however this is limited to seven heavy atoms. As the last few examples indicate, exhaustive combinatorial searches will be unable, for the foreseeable future, to deal with the molecular size relevant for organic electronics.

### b. Structural databases

As already alluded to, HTVS efforts which utilise databases of known chemical compounds have the advantage that any interesting compound found through them can be synthesised and is sufficiently stable for its initial characterisation to have taken place. Structural databases give additional access to the molecular geometry (within the crystal, Fig. 4b). For example, the CSD contains over 1 M stable crystalline entries with over 40 000 organic compounds with a relatively small computed HOMO–LUMO gap.^37^ In most cases, easy access to the work which outlines the synthesis is also made available. One immediate advantage of using these X-ray geometries is that the conformational search and energy optimisation step can be omitted with a substantial reduction of computational time. In our previous works to find SF^37^ and TADF^367^ candidates among the CSD, we could directly evaluate the low excited state energies and oscillator strengths on experimental geometries. Geometry optimisation was performed only on the most promising candidates computed as isolated molecules, most of which retained their desirable properties after optimisation. A. Stuke *et al.* also used the CSD for relevance and generated a dataset of 62 000 entries for spectroscopic applications;^368^ however, they relaxed the geometries using DFT, prior to computation of orbital energies.

It is particularly advantageous to use structural databases for HTVS when considering properties that depend on the intermolecular arrangement like charge mobility of excitonic properties. C. Schober *et al.* considered crystal arrangements to investigate high-mobility structures within the CSD.^269^ By considering a filtered set of approximately 95k crystal structures, they computed the electronic coupling between molecules in contact in the experimental geometry of the crystal and evaluated the mobility in the hopping limit. Another HTVS effort based on the CSD focused on the limit of coherent transport and included the effect of non-local electron phonon coupling.^50^ This required the evaluation of the local vibration of molecules embedded in their crystalline environment. Molecular arrangements in the crystal are also needed to study excitonic properties, as illustrated in a recent survey of ∼2200 crystals formed by molecules with bright lowest excited states for which the lowest excitonic band was characterised.^239^

### c. Chemical databases

In a similar vein to using geometries taken from structural databases, properties taken from chemical databases can also be used for HTVS. Chemical databases, such as PubChem^369^ and ZINC,^310^ enumerate millions of existing small molecules and have been heavily utilised in the field of drug discovery (a list of chemical databases is provided in ref. 370). The greatest advantage of using these databases is that each molecule is inherently synthesisable and often have chemical vendors linked for immediate purchase. Since experimental geometries are not provided, it is still necessary to construct reliable 3D geometries from the basic structural data encoded in the database, such as the very compact SMILES string (Fig. 4c).^371,372^ This means that, for a molecule, the usual geometry construction, conformer search and geometry optimisation should be performed prior to the computation of any specific property.

In their ML-based screening, P. M. Tagade *et al.* took a randomised subset of the PubChem database with approximately 78k structures to find a way of predicting redox potentials and frontier orbital energies based on chemical structure.^373^ They performed geometry optimisations for all of their structures at the B3LYP/6-311+G(d,p) level of theory. Following the same randomised selection logic, P. T. St. John *et al.* chose 40k closed-shell molecules, with 200k corresponding radicals, from the PubChem database for a DFT screening and provided their database for future ML works based on radical chemistry.^374^ However, in this case, they imposed criteria such as element selectivity, number of heavy atoms, net charge, bond type, *etc.* to ensure that all entries are relevant. This filtration is an important pre-step when using structural and chemical databases, especially for targeted properties. O. Borodin *et al.*, for example, considered only a specific set of 400 carbonate and phosphate molecules chosen from the PubChem database and screened for electrochemical stability of battery electrolytes.^375^

Considering much larger sets, the PubChemQC Project provides the ground-state electronic structure of 3M molecules from the PubChem database, and low-lying excited-state energies for 2 M of them *via* a web interface.^376^ Since the authors have performed calculation in increasing order of molecular weight, lighter molecules of more limited interest to organic electronics are more represented but the project well exemplifies the current capability of HTVS. In choosing the set of molecules to compute, molecules with erroneous representations in their cheminformatics representations (InChI^377^ and SMILES) were identified and removed, element selectivity based on the capability of the 6-31G* basis set were chosen and isotopes were ignored. All of the structures, having been generated using the Open Babel code,^378^ were subjected to optimisation using the semi-empirical PM3 method,^379,380^ Hartree–Fock with the STO-6G basis set, DFT at the B3LYP/6-31G* level and finally TD-DFT at the same level of theory.

### d. Generative models

**Fig. 5:**
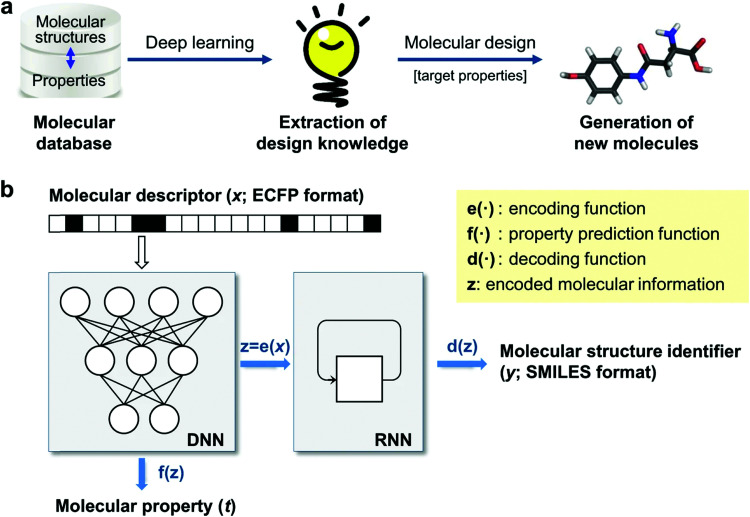
Data-driven inverse design. (a) Concept of inverse design: hidden knowledge for molecular design is extracted from a given molecular database in a fully data-driven manner using deep-learning, and new molecules with the target properties are generated subsequently. (b) Deep encoder–decoder architecture of inverse design model: the encoding and property prediction functions are obtained by a DNN using the molecular descriptor as an input, and the decoding function is obtained by an RNN using the encoding function as an input to generate the molecular identifier. Reprinted from ref. 388, under a Creative Commons Attribution 4.0 International (CC BY) license.

Generative models are trained with a database of molecules and their functionality and are then used to construct new chemical structures that have a desired functionality. This is often referred to as inverse design (see Fig. 5a), as opposed to the conventional approach where one first designs a compound and then predict its properties. Generative models have been used to generate novel information like new human faces^381^ or music,^382^ and some of these models, like variational auto-encoders^383^ and generative adversarial networks^384^ have recently been used to suggest prospective organic molecules.^385^ These models offer the possibility of aiding HTVS, as they can increase the amount of data analysed and offer an alternative to expand on human chemical intuition, with the potential to reduce human biases and detecting trends that may escape the human eye. Most applications of generative models in chemistry are in the field of drug discovery^386,387^ but these techniques have also been gaining traction in organic electronics.

In Fig. 5b, we show an inverse design strategy where a deep neural network (DNN) is used to encode the structural features and predict their target property, and a recurrent neural network (RNN) is used to decode this information and propose molecular structures with a desired target property. This approach has been used to construct an inverse design strategy that analysed 40 000 random chemical structures and was able to propose more than 3000 unique chemically plausible structures in the targeted range of T_1_ ≥ 3.0 eV, relevant for phosphorescent organic light-emitting diodes.^388^ Using DFT, it was found that 58.7% of the proposed molecules was in the desired range, which is a significant improvement from the 36.2% of molecules in the training set within that range. The correlation between T_1_ values predicted by the model and the DFT values was 0.881, and three of the proposed molecules were further validated experimentally, showing the potential for this type of inverse design strategies to target specific properties relevant to organic electronics. A similar approach has been used to construct generative and predictive models that were able to propose new non-fullerene acceptors whose properties were validated through DFT calculations.^389^ Other similar generative model has been used to propose new donor–acceptor oligomers with specific electronic properties, like the HOMO–LUMO gap and dipole moment, and it has been showed how the training data can affect the values of the electronic properties of the predicted oligomers.^390^

A critical point of generative models is the selection of the chemical space that is analysed. If one chooses a chemical space very similar to the one already known, one is merely interpolating known data. However, molecules that are too different from any known one can be risky to predict, as they can correspond to structures that are not chemically sensible or cannot be synthesised.^359,391^ It has been recently noted that generative models often ignore synthesisability. M. Sumita *et al.*^392^ prepared a platform to predict photofunctional organic molecules with excited states at a desired range using Monte Carlo tree search^393^ and a recurrent neural network. After just a few days of searching, the platform suggested 86 possible candidates, out of which five were confirmed to be synthesisable and stable. These results are promising, as it shows the potential of computer-aided chemistry to discover new molecules, but it also highlights how only a minority the predicted molecules can be synthesised if synthesisability is not explicitly considered when designing generative models, a problem already encountered in Section 3.a. W. Gao *et al.* used a data-driven computer-aided synthesis planning program to show that state-of-the-art generative models often result in non-synthesisable molecules,^357^ although this can be partially addressed by adding synthetic complexity heuristic to the models. In the last years, there have been several advances in computer-assisted synthesis planning (CASP) programs to overcome the lack of synthesisability prediction of generative models, by applying retrosynthetic transformations.^394–397^

## 4. Analysis

**Fig. 6:**
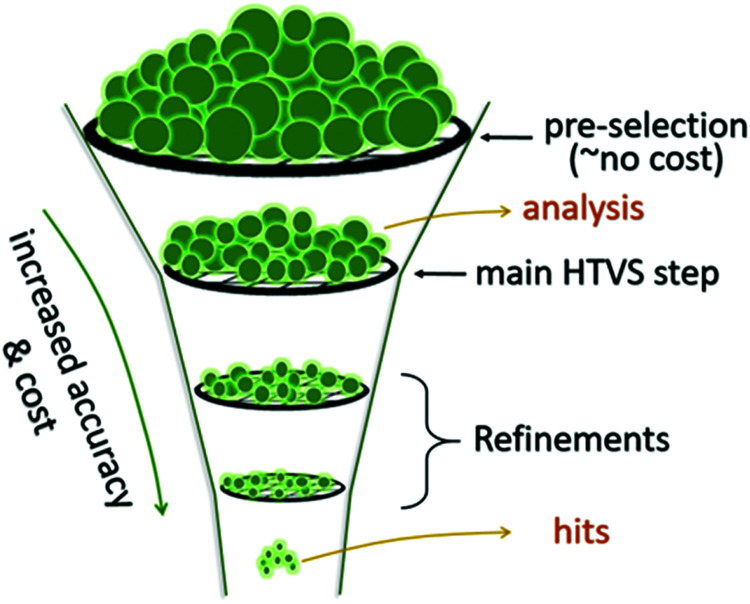
A revisited schematics of the computational funnel. The first and probably the most important step in is the definition of the systems to study and it normally has a negligible computational cost with respect to the rest. The first level of screening is often dominating the computational costs and it is the one that allows derivation of structure–property relations because of the size and homogeneity of the data produced. Refinements are sometimes needed but are less likely to give important insights.

Once an HTVS is completed, the large volume of data can be searched to find materials of a given property. The pool of potential candidates found based on preliminary criteria can be tightened by introducing refinement using higher levels of theory, along with a more rigorous selection of rules. After the number of candidates is reduced to include only the very best predicted by theory, experimentation can verify which, if any, of the small set of candidates can be used for device fabrication. This “computational funnel approach” popularised for example by Pyzer-Knapp *et al.*^24^ is not the only, or even the most common workflow, of HTVS. The pre-screening stage, where the systems to be studied are selected (Section 2 of this work) is always inexpensive. The first round of electronic structure computations is almost always the most expensive and produce a large set of homogenous data that is particularly precious for validating, disproving or discovering structure–property relations (Fig. 6). The following sections discuss how the results are HTVS can be analysed.

### a. Experimental verification of novel findings

Finding that a property is exhibited experimentally for molecules predicted by HTVS is the most important measure of success for a study seeking to find new optoelectronics. There are many barriers in the pipeline from theory to manufacturing such as chemical stability, environmental conditions and synthetic availability, therefore finding even one new material, among thousands, which can be used in a physical device can be considered as a major advancement. There have been a few attempts in the literature to directly combine HTVS with experimental testing, where some of the best candidates are shown to exhibit the desired property in a practical setting. A. Aspuru-Guzik and co-workers took this approach to seek molecules that exhibit TADF.^42^ They successfully found efficient materials, with a reported external quantum efficiency of over 22% in one, for potential use in devices by following their tiered screening approach. In a similar vein, they searched for molecules which exhibit SF by considering over 4000 anthracene derivatives,^36^ whereby anthracene is known to exhibit endothermic blue SF.^398,399^ Out of this set, they successfully found molecules with the desired energy levels, although with practical limitations.

Hund's rule has been questioned recently by the existence of singlet–triplet energy inversion in molecules such as heptazine.^400^ D. Miyajima *et al.* investigate this extremely novel property by combinatorial generation of a set of approximately 35 000 heptazine derivatives to find those with a negative singlet–triplet gap and with a sufficient oscillator strength, *i.e.* an emissive singlet state.^401^ After refinement of the best structures with highly correlated wavefunction methods, it was found that one such candidate exhibited delayed fluorescence from inverted singlet and triplet states experimentally. Seeking more traditional TADF molecules with a low singlet–triplet gap and those for SF with the opposite, T. Chen *et al.* investigated a set of triazines and the effect of their unique derivations on the singlet–triplet gap.^402^ With a congruent experimental and theoretical effort, they found that the impact of molecular symmetry and frontier orbital overlap can fine-tune the singlet–triplet splitting to sample a range which can accommodate both TADF and SF among the different triazines. Particularly intriguing is the combination of HTVS with high-throughput experimentation, two fields that are bound to converge.

A distinct but related form of validation is the rediscovery of molecules already known to have the desired property from a database that is not biased toward this property. A similar method was used to validate the screening of molecular semiconductors for SF,^37^ TADF^367^ and electron acceptors for OPV.^295^ In all these cases less than 0.5% of the screened materials had the desirable property but a fraction of them were already known to possess this property providing a good statistical validation of the procedure. There is no consensus among practitioners in the field on whether it is best to pursue experimental validation of HTVS within the same research report. On one hand, it is very useful to demonstrate the potential of the methodology through examples of success. On the other hand, experimental validations may introduce bias in the reporting of HTVS procedure. For example, there are virtually no joint prediction-experiment papers where the experiment disagrees with the prediction, *i.e.* the published literature may depict a too-optimistic view of the prediction accuracy.

It is worth making a connection with the area of autonomous chemical discovery where models and robotics synthesis are integrated.^403–405^ This approach is particularly valuable when libraries can be built on the basis of a limited number of precursors and synthetic conditions, *e.g.* donor–acceptor pairs. The virtual screening can be used to narrow down the number of precursors to consider and can become the first step of the autonomous discovery process. The best type of problems to be tackled with autonomous chemistry approach remains the optimisation of experimental conditions or composition of blends.^405^ However, the objective of HTVS is often the discovery of completely new lead compounds, requiring novel unpredicted precursors and synthetic routes. In these cases, HTVS and experimental validation cannot be easily integrated and will remain two separate stages of the discovery process.

### b. Validation of existing physical hypotheses and approximations

Supporting previous hypotheses and verifying the applicability of widely used approximations is one of the main outcomes of HTVS studies. For example, discovery of new SF materials has been driven by well-established and strict design rules based on biradicaloid structures,^165,166,406,407^ which has focused a narrow sight on the well-known acenes and only a few other classes. After expanding the library of predicted SF compounds,^37^ a follow-up screening which evaluated the diradical character of the potential candidates showed markedly that the biradicaloid characteristic was well-preserved among the vastly different molecules, but not all of them.^408^ Indeed, it is fairly frequent to discover exceptions to the established rules when large datasets are considered and occasionally one finds that the known rules are not valid in a statistical sense. For example, it seems that there is a complete lack of correlation between computed donor–acceptor character in a dye and its efficiency in a dye sensitised solar cell.^409^

In developing theories to describe charge transport in molecular semiconductors, often a number of different assumptions have been considered whose extent of validity can be verified using HTVS analyses. For instance, the analysis of ref. 18 on two structurally different materials extracted from the CSD confirms the viability of considering only a linear nonlocal electron–phonon coupling at least for the considered structures. Furthermore, it shows, in contrast to common assumptions, that the role of high-frequency modes in nonlocal electron–phonon coupling calculations cannot be completely ignored. For instance, the contribution of these modes to the fluctuation of the largest transfer integral of rubrene at room temperature is estimated to be 9%.^285^ In spite of strong coupling with high-frequency modes, the percentage is not remarkable because the high-frequency modes are not populated at room temperature. The other common assumption is that the valence band originates only from the HOMO orbital. An extended HTVS study on a set of ∼40 000 molecular semiconductors reveals that the median energy separation between HOMO and HOMO−1 energy levels is 0.66 eV whereas the charge transfer integral between the neighbouring molecules is never greater than 0.4 eV.^50^ This implies the band energies do not overlap effectively and, accordingly, this prevalent approximation is largely valid. The same study confirms the scarcity of molecular semiconductors with bands extending in three dimensions.

### c. Discovery of novel structure–property relations

Deriving insights from structure–property relations is an important strategy to develop new functional materials with tailored properties for organic electronics. The HTVS studies, designed to explore a large set of data, provide an excellent framework to derive novel approaches to materials discovery or to verify, in a statistically more rigorous way, the correlations that have been reported on limited samples in the literature. Confirming the lack of correlations between certain parameters is also another advantage of such analysis. The examples below illustrate these ideas in further detail.

**Fig. 7:**
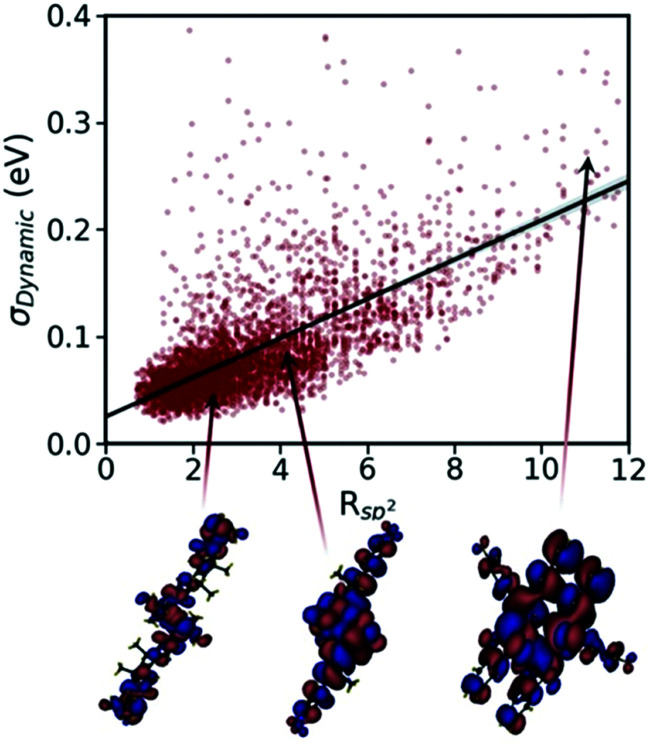
Correlation between dynamic disorder *σ*_Dynamic_ and fraction of sp^2^ atoms in contact (expressed in percentage) in molecular crystals: head to tail arrangement is surprisingly favourable for charge transport.

A frequent analysis of computed dataset involves the rationalisation of the coupling between electronic and nuclear degrees of freedom. An early example of this is the demonstration that the local electron–phonon coupling and the number of π bonded atoms are inversely proportional.^410^ In a more recent example, a large set of ∼5000 molecular semiconductors is screened to derive strategies for designing molecular crystals with a small level of dynamic disorder *σ*_Dynamic_.^411^ According to this analysis, the dynamic disorder between two molecules increases with the fraction of sp^2^-hybridised atoms in vdW contact, a property explainable with the nature of the overlap between molecular orbitals with many nodal planes (see Fig. 7). The very counterintuitive implication is that one should not try to design crystals with the maximal superposition of the molecular π-orbitals, as done for many years, because dynamic disorder is minimal when molecules are arranged in a “head-to-tail” configuration and their conjugated cores do not lie on the same plane. When the computed mobility itself is compared with the key system parameters,^50^ one finds a strong and expected correlation with the area per molecule, isotropy of the material and parameters controlling nonlocal and local electron phonon couplings and a relatively weak correlation with the transfer integral, in line with recent theories^265^ but not anticipated in the early days of organic electronics. Another interesting correlation is the recently reported link, in OPV cells, between electron–phonon coupling and non-radiative voltage losses for a large set of published and new material combinations data implying that the latter are, to some extent, unavoidable.^412^

Several works have attempted to identify the relation between luminescence and chemical structure. For instance, an extensive research incorporating cyclic voltammetry, thermogravimetric analysis, spectroscopic, and theoretical studies of spirobifluorene-derivatives reports that the molecular structure, conformational twisting, structural rigidity, and supramolecular packing play key roles in the photoluminescence of these molecules.^413^ Through generating a large set of molecular structures using ChemTS, a python library for *de novo* molecular generation,^414^ combined with a proper score function, the lack of correlation between the molecular shape and the high luminescence/absorption dissymmetry factor is reported.^415^ Structure–property relations have been sought in a database of 80 non-fullerene electron acceptors,^416^ finding that all high performing materials are characterised by a small gap between LUMO and LUMO+1 orbitals as proposed earlier on the basis of elementary models.^417^

The realisation of potential correlations between molecular structure and intra-/inter-molecular interactions has also been of interest. As such, a combined CSP and HTVS study on 27 structural isomers of pyrido[2,3-*b*]pyrido[3′,2′:4,5]pyrrolo[3,2-*g*]indole highlights the lack of correlation between molecular symmetry and preferred crystal packing and consequently intermolecular interactions.^307^ A recent work considering a set of over 2000 conjugated polyelectrolytes confirms known relations, *e.g.* that structures composed of alternating electron-rich and electron-poor heterocycle have lower bandgaps, and suggests several additional insights, *e.g.* that a more negatively charged anionic group and a shorter side-chain length are both correlated with higher HOMO level.^418^

### d. Determination of the physical limits of certain properties

Screening a large set of data can, not only, potentially identify a number of materials with targeted properties but also can be used to define the plausible physical limit to such properties. The latter is of particular technological interest as it determines how well one can expect to optimise each property. In performing such analysis in a recent project, through screening the CSD, our group identified the maximum achievable excitonic bandwidth (∼1.16 eV) and spectral red-shift (∼0.6 eV) in excitonic materials.^239^ In a similar analysis, a diverse set of conjugated molecules are evaluated to establish the relation between the exciton size and the size of the π-system.^419^ This analysis suggests that the exciton binding energy of ∼0.3 eV can be considered as the lower limit of this property which is in accordance with the typical theoretical data reported in the literature.^420^

**Fig. 8:**
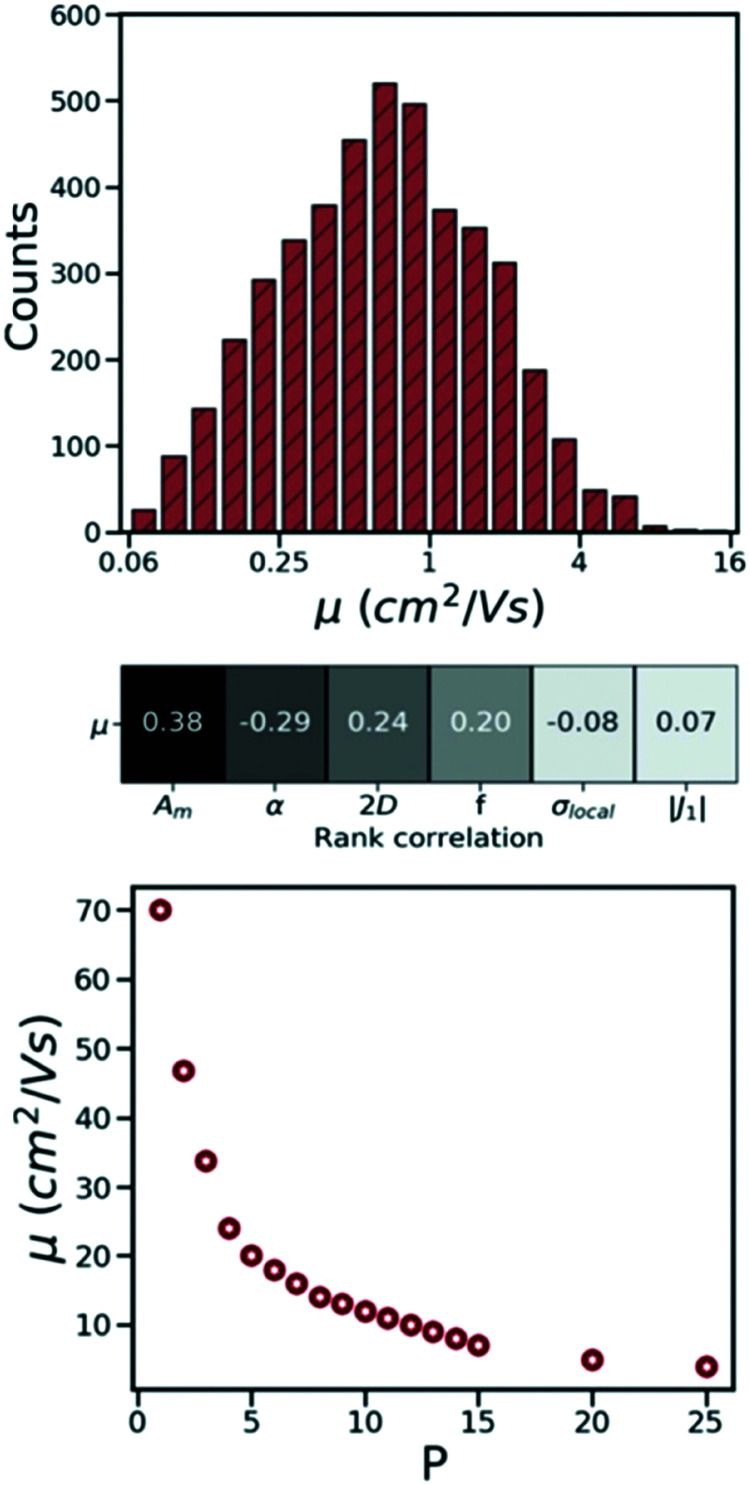
(top) Distribution of the computed mobility for a set of 5000 molecular semiconductors extracted from CSD. (middle) The rank correlation array between the mobility and important characteristics (molecular area *A*_m_, effective dynamic disorder *α*, transport two-dimensionality 2D, band renormalisation factor *f*, and the largest transfer integral *J*_1_). (bottom) Expected mobility for a hypothetical material with the important parameters being set simultaneously in the best *P* percentile of the distribution of real materials.

The largest plausible physical limit to charge carrier mobility in molecular semiconductors is evaluated through screening CSD as well.^50^ As such, confirming that the main parameters of high mobility materials are either uncorrelated or constructively correlated, a mobility of ∼70 cm^2^ V^−1^ s^−1^ (Fig. 8) is obtained for a hypothetical material where all its important parameters are set simultaneously to the best one percentile of the distribution found for real materials.

To find promising novel TADF emitters across the visible spectrum, in ref. 42, a large set of 400 000 molecules are screened using TD-DFT. The promising molecules are selected among those possessing high oscillator strength and low singlet–triplet gap and are synthesised and assessed experimentally to evaluate the predictive power of the employed screening protocol. The experimental measurements of the study, in agreement with theoretical predictions, record 22% as the highest electroluminescence quantum efficiency in the targeted molecules of the considered database.

Determining the limits of optical properties is also of great technological relevance. For instance, the transparency of millions of device configurations has been evaluated in an HTVS to identify the best semi-transparent photovoltaics device for power-generating windows to be used in buildings or automobiles.^421^ Accordingly, 11% PCE and 30% visible light transmittance are estimated as the upper limit of these structures’ characteristics. In another study, through applying the Lorenz–Lorentz equation to a set of over 60 polymers, the theoretical lower limit of the refractive index of organic polymers is evaluated to be 1.29.^422^

### e. Machine learning techniques to predict complex properties

**Fig. 9:**
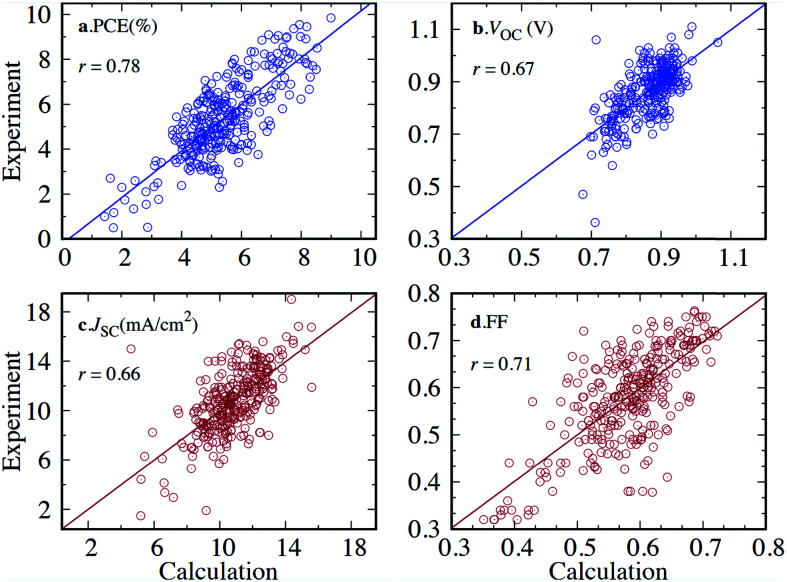
ML-predicted *versus* experimental data for PCE, *V*_OC_, *J*_SC_, and FF for all data points (300 OPVs) using the leave-one-out cross validation approach. Reprinted with permission from ref. 435. Copyright 2021 American Chemical Society.

Some complex properties, like the PCE of solar cells, cannot be computed from the knowledge of the chemical composition alone because of intrinsic limitations in the modelling capability, including an incomplete physical understanding of the underlying physics. Some simple models offer an approximation to these properties, for example, Scharber's model^423^ links the frontier molecular orbitals to the PCE, although it has been shown to have some limitations in reproducing recent experimental data.^424,425^ In these situations, ML models are especially useful and they can learn the correlations between the property of interest and specific features. For example, several studies have recently reported ML models able to predict PCE of OPVs with a large accuracy when using molecular fingerprints and a variety of physical features.^424,426–429^ These developments will not be reviewed in this work and we refer the reader to recent reviews on how ML has been applied to OPVs^430^ and energy materials as a whole.^431^ In typical situations, the ML model is a function that predicts experimental properties using, as the input, features of the systems that can be easily accessed. In organic electronic applications one often wishes to predict the properties of devices made with novel compounds for which there is no experimental information available. For this reason, many features used in ML methods are computed electronic structure parameters and a typical work identifies a functional relation between computed features and experimental figure of merit, sometimes followed by the generation of novel structures to be screened with the ML model (see, *e.g.*Fig. 9). Examples of ML guiding HTVS can be found in several fields like OPVs with fullerene acceptors^425^ and non-fullerene acceptors,^432^ polymer-fullerene solar cells,^433,434^ dye-sensitised solar cells,^40^ and organic light-emitting diodes.^42^

One should note that available datasets, especially in organic electronics, will likely have clusters of data points around known well-performing materials, while other areas of the chemical space will be sparsely populated also because of the reduced reporting of negative results. ML models, generally built for very large data sets, perform very poorly when trained on biased datasets. J. Sieg *et al.* have tried to tackle this problem and they have shown possible ways to correct for these biases,^436^ such as improved baseline assessments and scoring functions, or use higher quality/more abundant data. Some possible solutions to this data biases include the inclusion of failed experiments in the datasets,^437^ improved design of experiments^438^ and alternative metrics to judge the performance of ML models.^439,440^ Additionally, one should be careful when using ML to extrapolate the properties of new materials, *i.e.* calculating the properties of families of materials not present in the dataset, as their capacity to do so is contentious.^441,442^ Other recent studies have focused on how improving the ML-guided exploration of materials space, and for example active machine learning has proven able to identify OSCs with larger charge conduction properties than competing exploration strategies.^443^

Because of the way ML methods are conceived, they are unlikely to provide guidance on the maximum achievable performance on each technological domain as in the examples presented in Section 4.d but they can be very powerful in discovering novel correlations and ranking the importance of the features included in any model in particular with the advances of explainable ML.^439^ For example, recent studies have showed how ML can be a fast and efficient way to select effective molecular features correlated with the PCE of polymer materials,^444^ and can be used to rationalise the effects that the feature's trends have on the open-circuit voltage (*V*_OC_), short-circuit current (*J*_SC_) and fill factor (FF) of OPVs.^435^

## 5. Conclusions

As this review of the literature has shown, the application of the high-throughput screening method in organic electronics is constantly evolving and is now solidly a part of the digital discovery tools of molecular sciences. This evolution is driven by the ever-increasing number of applications where novel combinations of properties are required (see, *e.g.*Table 1), the improved understanding of the underlying physics (see *e.g.* Section 2.c), the acceleration in computational methodologies (see, *e.g.* Section 2.b), the collection of more robust datasets of materials and their properties (see, *e.g.* Section 2.a) and the ability to generate numerous and more efficient hypothetical materials to be explored (see, *e.g.* Section 3.d). The dynamicity of the field implies that it is very unlikely that any well-designed virtual screening protocol will provide the definitive answer for any given technological problem, not least because the scope of the screening can vary, *e.g.* from exploring all small variations of a given compound to considering a totally unexplored chemical space for breakthroughs. It is conceivable that each of the many quests for organic electronic materials will be accompanied for many years by a range of virtual screening protocols, just as it is the case for drug discovery. In this sense, being able to classify the different protocols in terms of a sequence of choices to be made and validated is helpful not only for navigating the literature but, more importantly, to introduce systematic improvements in the field.

Specific directions for improvement can be identified for all components of the protocol. Experimental datasets for validation should be expanded to cover a broader set of compounds of relevance for organic electronics using standardised characterization methodologies and, possibly, automation. Standardised computational datasets for the calibration of more approximate methods should be developed to accelerate the direct comparison of different approximate methodologies. The outcome of HTVS should be measured against novelty (with respect to common chemical knowledge) and feasibility (or cost effectiveness) of the discovered compounds. These are somewhat less quantitative aspects that nevertheless can be built in the construction of the virtual dataset to be explored and determine the impact of such explorations on technology.

Finally, high-throughput virtual screening will increasingly play a crucial role in the development of molecular science itself. It is, in many ways, the culmination of physical (or statistical) understanding where the degree of accuracy of the predictions is sufficient to determine the direction of future investigations and, in this sense, it can be seen as the ultimate objective of theoretical modelling. It also sets a new iteration in the process of scientific discovery with technologists exploiting the predictions validated by experiments and scientists focusing on what went wrong in the models and how to improve them. The large size of the datasets introduces, however, an important change to the traditional theory-experiment cycle. A well-executed high-throughput virtual screening does not allow the exclusion of predicted or experimental data points because, by definition, the data are too dense for individual inspection. The quantity of data prevents bias in the assessment of the quality of the model and this will unavoidably yield better models followed by better materials.

## Conflicts of interest

There are no conflicts to declare.

## Supplementary Material
